# Shift and night work during pregnancy and preterm birth—a cohort study of Swedish health care employees

**DOI:** 10.1093/ije/dyab135

**Published:** 2021-07-01

**Authors:** Manzur Kader, Carolina Bigert, Tomas Andersson, Jenny Selander, Theo Bodin, Helena Skröder, Mikko Härmä, Maria Albin, Per Gustavsson

**Affiliations:** 1 Institute of Environmental Medicine, Karolinska Institutet, Stockholm, Sweden; 2 Centre for Occupational and Environmental Medicine, Region Stockholm, Sweden; 3 Finnish Institute of Occupational Health, Helsinki, Finland

**Keywords:** Birth outcome, long shifts, pregnancy, preterm delivery, shift work, working hours

## Abstract

**Background:**

Previous studies of preterm birth (PTB) concerning night work have been inconclusive and partly limited by imprecise data on working schedules. This study investigated the risk of PTB in relation to detailed, registry-based data on working hours.

**Methods:**

In a register-based prospective cohort study, we identified 4970 singleton births with information on PTB from the Swedish Medical Birth Register of health care employees in Stockholm. Day-by-day information on working hours 2008–16 was obtained from a computerized employee register. Odds ratios (ORs) of PTB according to work hour characteristics were analysed by logistic regression adjusted for mother’s age, stature, body mass index (BMI), parity, smoking habits, education, profession and country of birth.

**Results:**

There was an increased risk of PTB among those who frequently worked night shifts (>25 times) [OR, 1.62; 95% confidence interval (CI), 1.03–2.53] and who ever worked ≥3 consecutive night shifts (OR, 1.43; 95% CI, 1.03–1.99) during the first trimester. Frequently (> 8 times) working 3 or more consecutive nights, and frequently (>18 times) having quick returns from night shifts (<28 h) during the first trimester showed 3–4 fold increased risk of PTB. Moreover, working frequent (>20 times) long shifts (≥10 h) (OR 1.63; 95% CI, 1.07–2.49) during the first trimester and working any Week >40 h (OR 2.05; 95% CI, 1.31–3.22) during the third trimester were associated with PTB.

**Conclusions:**

In this cohort of Swedish health care employees with registry-based data on working hours, night work, especially working frequent consecutive nights, and quick returns from night shifts during the first trimester were associated with increased risk of PTB among pregnant women.

Key Messages

- Using registry-based data on working hours among pregnant health care workers, night work, especially working frequent consecutive nights, and quick returns from night shifts were associated with an increased risk of preterm birth.
- Working long weeks (>40 h) during the third trimester was associated with the risk of preterm birth.
- Mothers whose first child was born preterm worked fewer nights during later pregnancies than did other mothers.
- Our findings imply that if pregnant women should work at night, few and single night shifts should be favoured, as well as allowing adequate recovery time after the night shifts.

## Introduction

The health care sector is composed mainly of female workers. Data from the Sixth European Working Conditions Survey (2017) showed that about 40% of women worked on shifts (irregular or unusual hours of work), and more than 15% of women worked longer than the standard 40-h workWeek .^1^ Disruption of the 24-h maternal circadian rhythm as a result of night work and long working hours can induce dysregulation of biochemical and neurophysiological functions, which may increase uterine contractility and the risk of preterm birth.^2^^,^^3^

Over the past decades, several studies investigated the relationship between working conditions, such as night work and long working hours, and adverse birth outcomes. Preterm birth (PTB), i.e. a gestational length of <37 completed weeks, is still considered to be the most important cause of perinatal mortality and morbidity.^4^ Some recent systematic reviews and meta-analyses have evaluated the evidence relating shift and night work and long working hours to adverse birth outcomes such as PTB.^5–9^ The overall evidence was found to be very weak, and the results have been inconsistent and inconclusive. This could result from heterogeneity between studies in terms of the study design and characteristics of the participants, as well as from differences in the assessment or definitions of shift or night work and long working hours. For example, in a review by Bonzini *et al*. (2007), working either shifts or nights was associated with a relative risk of >1.4 for PTB, compared with day work. In their updated reviews with more recent original studies, the association between shift work and PTB was found to be limited or small.^6^^,^^7^ A recent meta-analysis, with literature searches up to 15 March 2019, indicated that rotating shift work and fixed night shifts increased the risk of PTB by 13% and 21%, respectively, and that working >40 h per Week increased the risk of PTB by 21%.^9^ However, few studies were sufficiently statistically powered to investigate pregnancy period-specific analysis (first to third trimester). As the risk factors may play a different role in the different trimesters in relation to PTB, it is therefore not possible to compare the results of all studies together. Moreover, a majority of the previous cohort and case-control studies relied on self-reported information on working time and intensity of shift or night work. Further on, exposure information was often collected after the case status was known, which may result in recall bias.^10^ Therefore, further studies have been recommended to use detailed and more precise exposure information on working hours and the characteristics of the used shift work patterns.^11^ To avoid recall bias, a recent register-based cohort study in Denmark assessed working hours through payroll data in relation to PTB.^12^ In that study, night work in general was not associated with PTB.^12^

The present study includes detailed information on working hours from a computerized Human Resource (HR)-administrative system, called HEROMA, used in the health care sector of Region Stockholm (formerly ‘Stockholm County Council’). The method, using register-based working hours to classify working time patterns for different types of shift work, was successfully implemented in a study of Finnish health care workers and was recommended for the future.^13^ Consequently, the study aimed to investigate whether shift work or night work and long working hours during the first (Weeks 1–12), second (Weeks 13–28) and third trimester (Weeks 29–42) of pregnancy were associated with an increased risk of PTB. This was done based on the use of detailed and register-based individual information on working hours throughout the gestation.

## Methods

### Study design and population

This a prospective cohort study of pregnancies among health care professionals employed by Region Stockholm, from selected professions often working on shift and/or nights, that is nurses including midwives, nursing assistants and other related professions (e.g. accommodation assistants, careers, personal assistants). Physicians were not included in the present analysis due to less detailed information on working hours and night work.

Ethical permission for the study was granted by the Ethical Review Board in Stockholm (2016/2490–31; 2017/1157–32). Informed consent for participation in the study is not requested for register-based research.

### Data sources

The Swedish Medical Birth Register (MBR) provided data on outcome variables and some potential confounders (see below) that were collected at the initial interview in the first trimester at the prenatal care unit, typically at Week 10, and throughout the pregnancy and at childbirth, for all women and their children.^14^ The MBR was founded in 1973 and covers about 99% of all births in Sweden.^15^ It is one of the most complete birth registers in the world and has previously been validated. Its registered information on gestational age is considered to be reliable and quality is controlled on an annual basis.^15^

### Participants

We identified all births (*n *= 15 765) from the MBR concerning women who were employed any time between 2008 and 2017 and gave birth after the first day of employment. Out of these, 10 795 births were excluded due to any of the exclusion criteria: multiple births, *n* = 2067, professionals other than those of interest for the study, *n* = 2980, and births from women who were not employed during the whole pregnancy period, *n* = 5122, as well as births from women without work registration or undefined working hours, *n *= 626. Consequently, the present study included all singleton births (*n* = 4970) and their mothers (*n* = 3940) who were employed during their whole pregnancy period and had worked during at least one trimester of pregnancy at any time between 2008 and 2016. The reason for excluding women who terminated their employment during pregnancy was that their working hours could not be traced for the full pregnancy.

### Exposure

Information on working hours was retrieved from the employee register HEROMA used by Region Stockholm. The HEROMA contains detailed personal information on working hours day-by-day (number of minutes worked each hour) for all employees employed from 2008 and onwards, including information on occupation and workplace. Data for working time were aggregated from HEROMA to define work shifts for each trimester at each included pregnancy. A work shift should have a duration of at least 4 h to be called a shift. Three types of shifts were identified: day shift (starts after 06:00 and ends no later than 18:00); afternoon shift (starts after 12:00 and ends later than 18:00, but not a night shift); and night shift (at least 3 h of work within 22:00–06:00).^16^

### Basic night shifts and consecutive night shifts

For each trimester, we classified type of work schedules as ‘always day work’, ‘day and/or afternoon work (no nights)’, ‘day and/or afternoon work, and night work’, and ‘night work only’; and night work as ‘no night work’ and ‘night work ever’ (also including those who worked nights only) per trimester. Frequency of night shifts was categorized as ‘no night work’, ‘1–10 times’, ‘11–25 times’ and ‘>25 times’; consecutive night shifts as ‘1–4 times’, ‘5–8 times’ and ‘>8 times’, and the average number of nights in a row as ‘1–1.5 nights’, ‘1.6–2.5 nights’ and ‘>2.5 nights’ per trimester.

### Quick returns, duration of the shift, and long working week

We defined quick returns from night shifts as a recovery period of <28 h after a night shift, and quick returns from other shifts as a recovery period of <11 h after any other shift, in line with recent studies using payroll data.^12^^,^^13^ Categories of quick returns were ‘1–8 times’, ‘9–18 times’ and ‘>18 times’ per trimester. Long working for any shift (≥10 h) was classified as ‘no’, ‘1–10 times’, ‘11–20 times’ and ‘>20 times’, and long working weeks (working >40 h) as ‘no’, and ‘long week ever’ per trimester.

### Assessment of the outcome and demographic variables/covariates

The outcome of PTB was available through the MBR and was defined by a gestational length of <37 completed weeks.^14^ All the births, including stillbirths (*n* = 17) occurring at 22 completed weeks of pregnancy or later were considered for the study. The gestational length is most often defined by ultrasound early in pregnancy, preferably at 11–14 weeks, based on crown-rump and biparietal diameter.^14^

Demographic variables/covariates as potential confounders were identified based on a review of previous studies on risk factors for PTB.^4^ The demographic variables were mother’s age at childbirth, height, body mass index (BMI), parity and smoking habits at an initial interview at the prenatal care facilities at each pregnancy, obtained through the MBR. The highest level of the mother’s education at each included pregnancy was obtained from the longitudinal integration database for health insurance and labour market studies (LISA) at Statistics Sweden, used as a proxy for socioeconomic factors, and mother’s country of birth as a proxy measure for ethnicity categorized as Sweden, Nordic countries (except Sweden), Europe (except Nordic countries) and other countries.

### Data analysis

Baseline characteristics are presented as means (standard deviation: SD) for continuous variables and as percentages for categorical variables. Binary logistic regressions were used to estimate odds ratios (OR) with 95% confidence intervals (CI) for PTB in relation to the different dimensions of shift work and long working hours. Analyses were adjusted for the potential confounders. Some analyses were also adjusted for the number of total night shifts among those who worked at least one night shift during the trimester, in order to disentangle the effect of working consecutive night shifts or quick returns from the intensity of night work in general. We performed comparisons of different dimensions of night shift work with non-night shift work, and comparisons within those with at least one night shift during any trimester where the night shift workers with the lowest exposure category served as the reference group. The latter was done to strengthen the evidence of a causal effect, as the category of night shift workers is more homogeneous and may be less susceptible to systematic selection from night work. To capture uncontrolled confounding by any previous negative birth experience, we also applied sensitivity analyses. First, the analysis was delimited to first-time pregnant women. Secondly, we investigated if a PTB at first pregnancy influenced night work during second or later pregnancies, by comparing the average number of night shifts in mothers with the first child born full-term with the average number of night shifts in mothers whose first child was born preterm.

The statistical analyses were conducted using SAS software, version 9.4 for Windows (SAS Institute Inc., Cary, NC, USA), with the statistical association at a two-tailed *P* value below 0.05.

## Results

**Figure 1:**
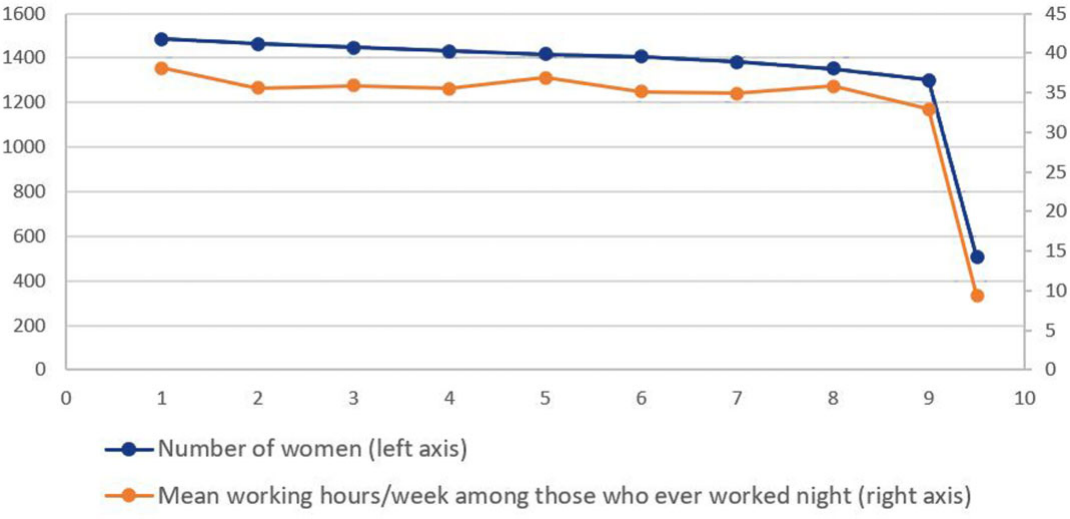
Number of working women and average working hours/week per month of gestation among those who ever worked nights

The baseline characteristics of the study participants in relation to work schedule are presented in Table 1. The mean [standard deviation (SD)] age of the women was 32 (4.4) years, with a mean age (SD) of 1.9 (0.9) of children. The mean (SD) birthweight of the infants was 3540 (±566) g, and 257 (5.1%) of the infants were born preterm. In total, 18% of the employees worked day shifts only, 52% worked at least one afternoon shift and 30% at least one night shift during the whole pregnancy. Compared with day workers, shift workers, with or without night shifts were younger, more often smokers and had fewer children. Moreover, nursing assistants more often worked on shift with or without night shifts than the nurses (Table 1). In general, very few women in the study were smokers (*n* = 98, 2%). Among all the women who worked at least one night shift during pregnancy, 1484 women worked in the first month of gestation, which decreased to 1301 women at the ninth month of gestation (Figure 1).

**Table 1:** Baseline characteristics of the study participants^a^ with singleton births (*n* = 4970) by work schedule, 2008–16

	Day work^b^			Shift work, without night shift^c^			Shift work, with night shift^d^		
	*n* = 906			*n* = 2580			*n* = 1484		
Variables	*n*	(%)	Mean (SD)	*n*	(%)	Mean (SD)	*n*	(%)	Mean (SD)
Age									
<25 years	21	2.3		192	7.4		91	6.1	
26–30 years	185	20.4		943	36.6		556	37.5	
31–35 years	380	42.0		965	37.4		544	36.7	
>35 years	320	35.3		480	18.6		293	19.7	
Height	894		166.8 (6.3)	2526		166.5 (6.2)	1459		166.9 (6.3)
Missing	12			54			25		
Body mass index, kg/m^2^			24.1 (4.3)			24.0 (4.0)			24.5 (4.5)
Missing	28			125			59		
Parity									
1	271	29.9		1095	42.4		690	46.5	
2	380	41.9		964	37.4		525	35.4	
≥3	255	28.2		521	20.2		269	18.1	
Smoking at enrolment									
Non-smokers	889	99.1		2478	97.6	97.8	1440	98.0	
Smokers	8	0.9		61	2.4	2.2	29	2.0	
Missing	9			41			15		
Education									
Higher education (university ≥3 years)	727	80.9		1856	72.4		1234	83.9	
Upper secondary/ elementary or less	172	19.1		707	27.6		238	16.1	
Missing	7			17			12		
Country of birth									
Sweden	753	83.1		2111	81.8		1224	82.5	
Nordic countries (except Sweden)	25	2.8		58	2.3		49	3.3	
Europe (except Nordic countries)	17	1.9		65	2.5		49	3.3	
Other countries	111	12.2		346	13.4		162	10.9	
Profession									
Nurses including midwives	683	75.4		1664	64.5		1103	74.3	
Nursing assistants^e^	223	24.6		916	35.5		381	25.7	

a Restricted to births among the women who worked full time or part time during their whole pregnancy period.

b Day work: starts after 06.00 and ends no later than 18.00.

c At least one afternoon shift (starts after 12.00 and ends later than 18.00, but not a night shift).

d At least one night shift (at least 3 h of working shift within 22:00–06:00), also includes women who work night shifts only.

e Nursing assistants: assistant nurses, carers, accommodation assistants, personal assistants etc.

### Basic shift characteristics, consecutive night shifts, and PTB

Women who ever worked the night shift during the first trimester had a slightly increased risk of PTB [adjusted OR (aOR) 1.32; 95% CI, 0.98–1.76], compared with no night work. Women who worked the night shift frequently (>25 times) during the first trimester were 1.62 times more likely to give birth to an infant with PTB (aOR, 1.62; 95% CI, 1.03–2.53) compared with those with no night shifts. No associations were observed in the analysis of the second and third trimesters, except for working night shifts 1–10 times during the third trimester (aOR, 1.57; 95% CI, 1.03–2.39); (Table 2). Among first-time mothers, associations were present also for trimesters two and three, see below.

**Table 2:** Odds ratios of preterm birth^a^ in relation to basic shift characteristics and consecutive night shift work during pregnancy among health care professionals,^b^ 2008–16

	Preterm birth, trimester one		Preterm birth, trimester two		Preterm birth, trimester three	
Variables	*n*, non-cases/ cases	Adjusted OR^**c**^ (95% CI)	*n*, non-cases/ cases	Adjusted OR^**c**^ (95% CI)	*n*, non-cases/ cases	Adjusted OR^**c**^ (95% CI)
Work schedule, per trimester						
Always day work	1012/51	Ref.	1023/51	Ref.	1141/56	Ref.
Day and/or afternoon work (no nights)	2378/117	0.97 (0.68–1.39)	2386/126	1.05 (0.74–1.50)	2491/119	0.96 (0.68–1.36)
Day and/or afternoon work, and night work	930/63	1.30 (0.87–1.95)	929/59	1.23 (0.82–1.86)	679/41	1.19 (0.76–1.85)
Night work only	227/14	1.26 (0.68–2.35)	208/14	1.44 (0.77–2.68)	213/14	1.42 (0.76–2.63)
Any night work during trimester						
No	3390/168	Ref.	3409/177	Ref.	3632/176	Ref.
Yes	1157/77	1.32 (0.98–1.76)	1137/73	1.23 (0.92–1.65)	892/55	1.27 (0.92–1.76)
Frequency of night shifts						
No night work	3390/168	Ref.	3409/177	Ref.	3632/176	Ref.
1–10 times	541/36	1.28 (0.86–1.91)	537/32	1.15 (0.77–1.72)	396/32	1.57 (1.03–2.39)
11–25 times	306/16	1.05 (0.60–1.82)	246/18	1.35 (0.79–2.30)	202/15	1.63 (0.93–2.83)
>25 times	310/25	1.62 (1.03–2.53)	354/23	1.26 (0.79–2.00)	294/8	0.61 (0.29–1.25)
Type of consecutive night shifts						
No night work	3390/168	Ref.	3409/177	Ref.	3632/176	Ref.
Only single night shifts	82/4	0.78 (0.24–2.51)	93/5	1.19 (0.47–2.99)	57/4	1.72 (0.61–4.86)
Up to two consecutive night shifts	320/19	1.18 (0.70–1.98)	304/17	1.07 (0.63–1.83)	218/13	1.09 (0.58–2.05)
≥3 consecutive night shifts	755/54	1.43 (1.03–1.99)	740/51	1.29 (0.92–1.82)	617/38	1.29 (0.89–1.89)
Frequency of ≥3 consecutive night shifts						
1–4 times	531/32	Ref.	434/34	Ref.	376/29	Ref.
5–8 times	150/11	1.42 (0.66–3.03)	199/10	0.75 (0.342–1.64)	150/5	0.54 (0.20–1.48)
>8 times	74/11	3.01 (1.38–6.55)	107/7	0.93 (0.38–2.24)	91/4	0.61 (0.20–1.87)
Frequency of ≥3 consecutive night shifts (additionally, adjusted for no. of night shifts)						
1–4 times	531/32	Ref.	434/34	Ref.	376/29	Ref.
5–8 times	150/11	1.76 (0.69–4.46)	199/10	0.42 (0.16–1.11)	150/5	1.34 (0.35–5.09)
>8 times	74/11	4.16 (1.35–12.82)	107/7	0.38 (0.11–1.33)	91/4	2.12 (0.40–11.00)
Average number of nights in a row						
1–1.5 nights	186/6	Ref.	196/10	Ref.	107/6	Ref.
1.6–2.5 nights	749/52	2.92 (1.03–8.25)	729/47	1.15 (0.56–2.36)	576/35	1.27 (0.47–3.38)
>2.5 nights	220/19	4.03 (1.33–12.20)	209/16	1.39 (0.60–3.21)	206/14	1.36 (0.46–4.05)
Average number of nights in a row (additionally, adjusted for no. of night shifts)						
1–1.5 nights	186/6	Ref.	196/10	Ref.	107/6	Ref.
1.6–2.5 nights	749/52	2.74 (0.94–8.00)	729/47	1.02 (0.47–2.19)	576/35	1.65 (0.60–4.52)
>2.5 nights	220/19	3.68 (1.14–11.86)	209/16	1.16 (0.46–2.92)	206/14	1.87 (0.61–5.72)

a Restricted to the births of women employed full time or part time during their whole pregnancy period, and preterm birth was dichotomized as <37 weeks and ≥37 weeks.

b Nurses including midwives, and nursing assistants, (e.g. assistant nurses, carers, accommodation assistants, personal assistants).

c Adjusted for mother’s age [25–30 years (reference), <25 years, 31–35 years, >35 years], mother’s height, BMI, parity [1 (reference), 2, ≥3], smoking habits [yes, no (reference)], education [higher education (university ≥3 years) (reference), upper secondary/elementary or less], country of birth [Sweden (reference), Nordic countries (except Sweden), Europe (except Nordic countries), other countries], and profession [midwives/nurses (reference), nursing assistants].

However, smoking was not adjusted for 2 and ≥3 consecutive night shifts and average number of nights because of missing data of smoking for cases in some categories.

Women who ever worked three or more consecutive night shifts during the first trimester were 43% more likely to give birth to an infant with PTB (aOR, 1.43; 95% CI, 1.03–1.99); compared with those with no night work during that trimester. The risk for PTB was higher (aOR, 3.01; 95% CI, 1.38–6.55) among those who frequently (>8 times) worked three or more consecutive night shifts during the first trimester, compared with those who did so 1–4 times. Working on the average 1.6–2.5 nights and >2.5 nights in a row during the first trimester was associated with a 3–4-fold increased risk of PTB compared with working 1–1.5 nights in a row. After adjusting for the total number of night shifts, the associations remained for three or more consecutive night shifts and for the average number of nights during the first trimester, although with wider confidence intervals (Table 2).

### Quick returns, duration of the shift and long working week, and PTB

Women who frequently (>18 times) had quick returns from night shifts during the first trimester had an increased risk for PTB (aOR, 2.42; 95% CI, 1.23–4.75), compared with those with quick returns 1–8 times. After adjusting for the total number of night shifts, more frequent quick returns from night shifts (9–18 times, and >18 times) during the first trimester was associated with PTB, compared with quick returns 1–8 times (Table 3). Increased aORs were observed for PTB in women working long shift (≥10 h) frequently (>20 times) during the first trimester (aOR, 1.63; 95% CI, 1.07–2.49), and working 1–10 times during the third trimester (aOR, 1.66; 95% CI, 1.16–2.35) compared with those with no longer shifts. Moreover, working any week for >40 h during the third trimester was associated with a more than 2-fold increased risk of PTB (aOR, 2.05; 95% CI, 1.31–3.22) (Table 3).

**Table 3:** Odds ratios of preterm birth^a^ in relation to quick returns, and long working hours and long weeks during pregnancy among health care professionals,^b^ 2008–16

	Preterm birth, trimester one		Preterm birth, trimester two		Preterm birth, trimester three	
Variables	*n*, non-cases/ cases	Adjusted OR^c^ (95% CI)	*n*, non-cases/ cases	Adjusted OR^c^ (95% CI)	*n*, non-cases/ cases	Adjusted OR^c^ (95% CI)
Frequency of quick returns from night shift (<28 h)						
1–8 times	632/37	Ref.	594/34	Ref.	450/32	Ref.
9–18 times	326/22	1.38 (0.77–2.48)	230/21	1.79 (0.97–3.29)	191/14	1.37 (0.69–2.75)
>18 times	141/15	2.42 (1.23–4.75)	241/15	1.41 (0.72–2.73)	204/5	0.48 (0.18–1.31)
Frequency of quick returns from night shift (<28 h) (additionally, adjusted for no. of night shifts)						
1–8 times	632/37	Ref.	594/34	Ref.	450/32	Ref.
9–18 times	326/22	3.33 (1.17–9.41)	230/21	2.25 (0.79–6.36)	191/14	4.39 (1.30–14.78)
>18 times	141/15	11.51 (2.09–63.30)	241/15	2.17 (0.39–12.02)	204/5	4.41 (0.49–39.11)
Frequency of quick returns from other shifts (<11 h)						
1–8 times	1118/53	Ref.	931/42	Ref.	1010/80	Ref.
9–18 times	1554/93	1.36 (0.94–1.98)	1543/88	1.34 (0.90–1.98)	1199/51	0.55 (0.38–0.80)
>18 times	262/13	1.21 (0.64–2.28)	449/26	1.31 (0.77–2.23)	578/3	0.07 (0.02–0.22)
Long work shifts (≥10 h) (any shift)						
No long shifts	3085/152	Ref.	3095/167	Ref.	3422/161	Ref.
1–10 times	819/49	1.16 (0.82–1.64)	816/40	0.95 (0.66–1.36)	601/49	1.66 (1.16–2.35)
11–20 times	275/15	1.01 (0.56–1.81)	230/14	0.94 (0.50–1.77)	173/9	1.14 (0.57–2.30)
>20 times	368/29	1.63 (1.07–2.49)	405/29	1.37 (0.90–2.09)	328/12	0.86 (0.47–1.57)
Long working weeks (>40 h) ever (all shift workers)						
No long working weeks	4078/225	Ref.	4180/229	Ref.	4252/203	Ref
Yes	453/19	0.77 (0.47–1.25)	350/21	1.04 (0.64–1.69)	255/26	2.05 (1.31–3.22)

a Restricted to the births of women employed full time or part time during their whole pregnancy period, and preterm birth was dichotomized as <37 weeks and ≥37 weeks.

b Nurses including midwives, and nursing assistants, (e.g. assistant nurses, carers, accommodation assistants, personal assistants).

c Adjusted for mother’s age [25–30 years (reference), <25 years, 31–35 years, >35 years], mother’s height, BMI, parity [1 (reference), 2 ≥ 3], smoking habits [yes, no (reference)], education [higher education (university ≥3 years) (reference), upper secondary/elementary or less], country of birth [Sweden (reference), Nordic countries (except Sweden), Europe (except Nordic countries), other countries], and profession [midwives/nurses (reference), nursing assistants].

However, smoking was not adjusted for quick returns from night shift and other shift because of missing data of smoking for cases in some categories.

### Changes of work schedule per month of gestation

**Figure 2:**
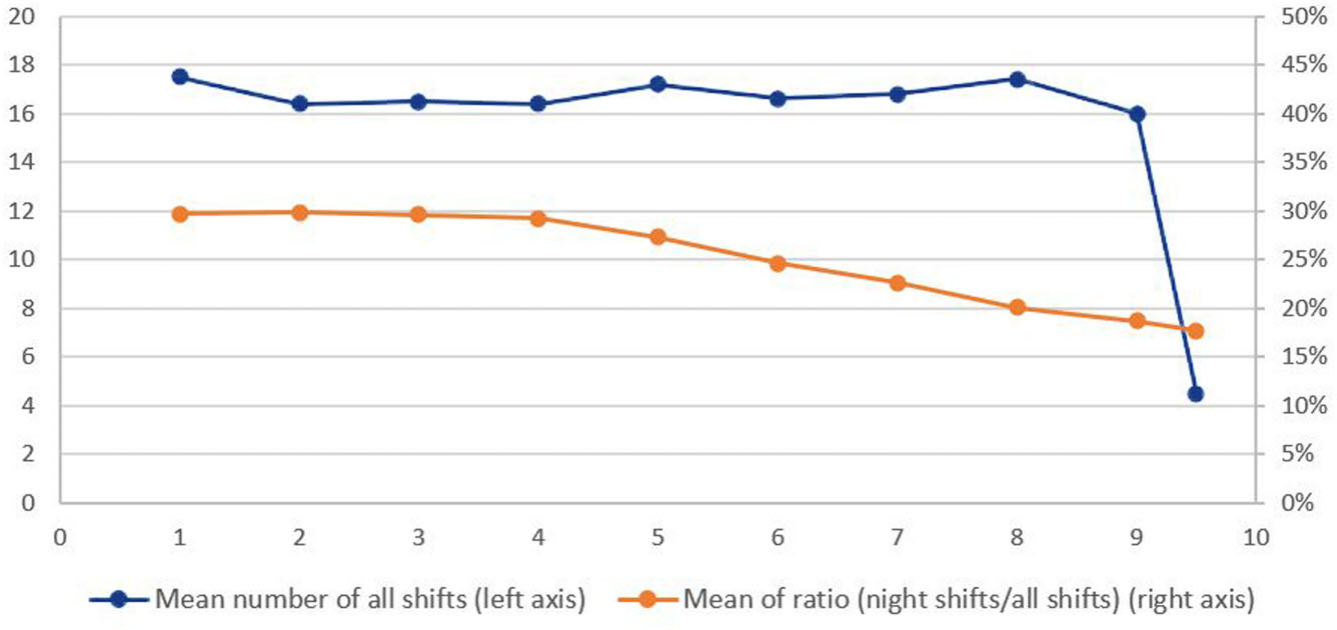
Number of shifts and proportion of night shifts per month of gestation among those who ever worked nights

**Figure 3:**
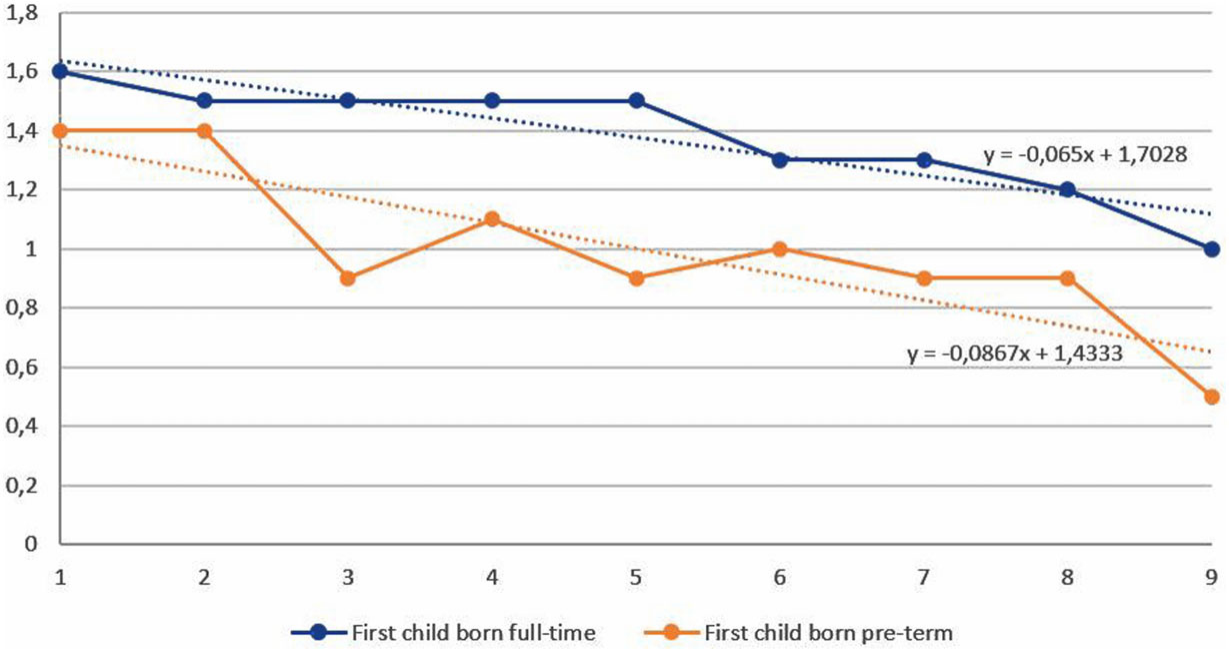
Average number of night shifts during second or later pregnancy among women with first child born full term or preterm

There was a decrease in mean working hours per week (from 38.1 to 32.9 h) from the first month to the ninth month of gestation (Figure 1). Although there was no remarkable decrease in the mean number of all shifts (from 17.5 to 17. 4 shifts), there was a decrease in the mean ratio of night shift work (from 30% to 19%) at the first month gestation to the ninth month of gestation (Figure 2). Thus, there was a gradual transition to day work during pregnancy, and this transition started already in trimester two (Figure 2). A preterm birth at first pregnancy was consistently associated with less night work at later pregnancies, both at the beginning and during the course of pregnancy (Figure 3). In the first month of the second or later pregnancies, women with a first child born full term worked on the average 1.6 nights per month, whereas mothers with a first child born preterm worked 1.4 nights, and this difference persisted throughout pregnancy. Both the difference in the average number of night shifts and the ratio between night shifts in the two groups tended to increase during the course of pregnancy. The difference increased by 6.2% per month of gestation in a linear regression model (beta = 0.062, *P *=* *0.10). The ratio of the average number of night shifts per month among mothers with and without previous PTB decreased from around 0.9 in the first month by a factor of 0.03 per gestational month, in a linear regression model (beta = 0.03, *P *=* *0.08) (Figure 3). 

### First-time pregnancies

Sensitivity analyses of first-time pregnant women (*n* = 2056) yielded broadly similar results as the main analyses for trimester one, although with a wider confidence interval. One important difference was that many aspects of night work were associated with increased risks also in trimesters two and three, and some risk associations in trimester one were slightly higher among the primiparous women than among all pregnant women. For example, working ever three or more consecutive nights during the second and/or third trimester were associated with PTB (aOR, 1.60; 95% CI, 1.01–2.54 and aOR, 1.71; 95% CI, 1.04–2.82, respectively) (Supplementary Table S1, available as Supplementary data at *IJE* online), which was not the case in the main analyses.

## Discussion

This cohort of Swedish health care employees using detailed individual information on working hours per trimester found that the risk of PTB was associated with working a higher number of night shifts, consecutive night shifts and long shifts (≥10 h) as well as frequent quick returns (<28 h) from night shift during the first trimester, after adjustment for all available potential confounders. Working long weeks (>40 h) during the third trimester was also associated with the risk for PTB. The associations remained after adjusting for the total number of night shifts. We furthermore found that the mean ratio of night shift work decreased and non-night work gradually increased with the progression of gestation, which indicates the phenomenon of ‘healthy worker effect’ where employees are selected or self-selected out of night shift work due to poor health or more pregnancy complications.^17^ Our data confirmed that mothers who earlier had had a baby born preterm worked fewer nights in later pregnancies, and the difference to other mothers tended to increase throughout pregnancy. Our findings of a higher risk of PTB associated with exposure to night shift work during the first trimester and lack of effect in later trimesters in the full dataset may partly be explained by this systematic selection from night work. The analyses restricted to first-time pregnancies corroborated this by showing negative effects of night work also in trimesters two and three (although with wider confidence intervals).

In many cases, the analyses were limited to the night shift workers only with the lowest exposure category serving as the reference group. These findings strengthen the evidence of a causal effect, as the category of night shift workers is more homogeneous and may be less susceptible to systematic selection from exposed work. These findings may therefore be more appropriate from an epidemiological point of view. After we added the total number of night shifts in the model when investigating effects in night shift workers, a majority of the associations with PTB became stronger than before during the first trimester. These findings also show that the association is not present just because workers with many consecutive night shifts in general have worked more night shifts than those with fewer consecutive night shifts. This suggests that more nights are more harmful than few nights, but with the same number of nights it is more harmful if these are consecutive nights. Our finding of a lower risk for PTB during the third trimester associated with a high frequency of quick returns from other shifts remain unexplained, but the results for >18 quick returns was based on only three exposed cases. This could be a chance finding, and a similar phenomenon was also seen in a previous study.^13^

To our knowledge, there is only one previous study from Denmark which investigated different dimensions of night work through registered-based data (through payroll system) in relation to PTB, where night work in general was not found to be associated with PTB.^12^ Our findings of increasing odds ratios of PTB with increasing exposure to night shift work and long working hours may relate to circadian rhythm disturbances, which pose a significant challenge to pregnant women and their fetuses through changes in physiological and behavioural rhythms such as hormone secretions, vascular stress and sleep disorders.^2^^,^^3^^,^^18–20^ Several experimental studies found that the number of consecutive night shifts has been associated with progressive changes in hormones involved in circadian rhythms, such as melatonin and cortisol.^21–24^ Previous studies have observed that three or more consecutive night shifts were associated with a decrease in melatonin among female hospital employees,^22^ and among both males and females in the general population^23^ in Canada, as well as among female nurses in Denmark.^24^ Furthermore, it has been suggested that nurses must be allowed more than 2 days off work after changing from night shift to other shifts.^25^ Our findings support the potential effect of insufficient recovery after a night shift.

Working long weeks only in the third trimester was associated with the risk of preterm birth. The mechanisms behind the effects of long working weeks and night work may differ and thus have different impacts on the risk of PTB in different phases of pregnancy. A plausible physiological mechanism might be the increase in the release of noradrenaline and catecholamine due to long working hours during the third trimester, which may increase uterine contractility and the risk of PTB.^26^^,^^27^

### Clinical implications

Shift work, including night work and long working hours, during pregnancy is an exposure of concern according to the occupational safety regulations for pregnant women in the EU. The employer is obliged to make a risk assessment, but this is hampered by a lack of knowledge of which specific conditions in long work or night work constitute a risk.^28^ Thus, our study will contribute to better understand how to organize shift work during pregnancy to avoid or to minimize the risk. One strength of our study is that it includes women in the health care sector where the percentages of shift and night workers are high. Thus, our study will contribute to better understanding of the challenges and to developing solutions on how the health sector can offer better quality employment to women. Our study will guide us to find preventive measures against negative pregnancy outcomes and will contribute to policy making regarding working hours during pregnancy.

### Strengths and limitations

The main strengths of our study include using a large and prospective cohort dataset with registered-based exposure information in an employee register. The data provided a unique opportunity to study shift work in more detail with several dimensions of the working hours or schedule specific to each trimester. All exposure information was collected before the outcome information was known. The outcome and confounders from the MBR and LISA are of high quality, with few missing observations. Information on the outcome, exposures and confounders was obtained at each pregnancy. With prospectively collected data, recall bias does not constitute a problem in our study. Another strength is that our study population represents a homogeneous group of health care employees with less variability in potential confounders related to socioeconomic position.

Our study had some limitations. We did not have information on physically demanding workload (e.g. heavy lifting, prolonged standing) during pregnancy, which was found to be associated with PTB in previous studies.^29^ However, the adjustment for occupation should have tended to decrease this source of error. Likewise, we had no information on exposure to anaesthetics or chemotherapeutics, but both these exposures would tend to be more common among day-time than among night-time workers. We did not have information on the sleep, chronotype and personal preferences of the participants. It was earlier found that women with morning chronotype tend to find night shifts more strenuous compared with women with evening chronotype.^30^ Our study includes only health care employees, with the majority having irregularly changing schedules nearly weekly. Therefore, our results may not apply to populations with other occupations.

## Conclusions

In this cohort of Swedish health care employees with registered-based data on working hours, night work, especially working frequent consecutive nights and quick returns from night shifts during the first trimester, and working long weeks during the third trimester, were associated with increased risk of PTB in pregnant women. Risks for PTB in association with night work were slightly stronger among the primiparous mothers, and our analyses showed a selective and systematic transferral from night work in mothers who previously had a child born preterm. This transferral may have contributed to the apparent lack of negative effects of night work in trimesters two and three. Our study can help women and employers to make more informed decisions when it relates to shift work and long working hours during pregnancy. A recent position paper recommended that pregnant women should work no more than one night shift in a week .^31^ Our findings show that if pregnant women work at night, few and single night shifts should be favoured, as well as allowing adequate recovery time after the night shifts. Long working hours should be avoided, especially during the third trimester. Ideally, future studies on PTB and night work during pregnancy should combine objectively measured working hours with information on physical and mental workload, chronotype and personal preferences, and should include other occupational groups.

## Data availability

The sensitive and confidential data used in this study cannot be made public, according to the General Data Protection Regulation, the Swedish law SFS 2018:218, the Swedish Data Protection Act, the Swedish Ethical Review Act and the Public Access to Information and Secrecy Act.

## Supplementary Data

Supplementary data are available at *IJE* online.

## Funding

This study was supported by grant from Swedish Research Council for Health, Working life and Welfare (No 2016–00748).

## Supplementary Material

dyab135_Supplementary_Data
