# Supplementary material for: Shift and night work during pregnancy and preterm birth—a cohort study of Swedish health care employees
Source: Int J Epidemiol. 2021 Jul 1;50(6):1864–74. doi: 10.1093/ije/dyab135 (PMC8743126; doi:10.1093/ije/dyab135)
Supplement: dyab135_Supplementary_Data [file dyab135_supplementary_data.docx]

**Table S1**. Odds ratios of preterm birth^a^ restricted to first-time pregnant women^b^ only (N=2056).

|  | Preterm birth, Trimester one | |  | Preterm birth, Trimester two | |  | Preterm birth, Trimester three | |
| --- | --- | --- | --- | --- | --- | --- | --- | --- |
| Variables | N,  Non-cases /cases | Adjusted OR^c^ (95% CI) |  | N,  Non-cases /cases | Adjusted OR^c^ (95% CI) |  | N,  Non-cases /cases | Adjusted OR^c^ (95% CI) |
| Work schedule, per trimester |  |  |  |  |  |  |  |  |
| Always Day work | 307/16 | Ref. |  | 312/17 | Ref. |  | 369/21 | Ref. |
| Day and/or afternoon work (no night) | 1021/57 | 1.16 (0.63–2.11) |  | 1050/64 | 1.28 (0.71–2.32) |  | 1134/63 | 1.07 (0.62–1.85) |
| Day and/or afternoon work, and night work | 469/44 | **1.89 (1.01**–**3.53)** |  | 442/38 | 1.74 (0.92–3.29) |  | 292/30 | **1.91 (1.03–3.55)** |
| Night work only | 70/4 | 1.15 (0.36–3.64) |  | 58/5 | 1.91 (0.65–5.55) |  | 65/5 | 1.45 (0.51–4.11) |
| Any night work during trimester |  |  |  |  |  |  |  |  |
| No | 1328/73 | Ref. |  | 1362/81 | Ref. |  | 1503/84 | Ref. |
| Yes | 539/48 | **1.59 (1.06**–**2.37)** |  | 500/43 | 1.44 (0.96–2.17) |  | 357/35 | **1.72 (1.11–2.66)** |
| Frequency of night shifts |  |  |  |  |  |  |  |  |
| No night work | 1328/73 | Ref. |  | 1362/81 | Ref. |  | 1503/84 | Ref. |
| 1–10 times | 284/25 | 1.47 (0.88–2.45) |  | 265/20 | 1.26 (0.73–2.16) |  | 180/26 | **2.55 (1.54–4.20)** |
| 11–25 times | 154/9 | 1.17 (0.57–2.42) |  | 118/12 | 1.70 (0.87–3.33) |  | 83/6 | 1.32 (0.55–3.16) |
| >25 times | 101/14 | **2.51 (1.32**–**4.77)** |  | 117/11 | 1.60 (0.79–3.23) |  | 94/3 | 0.58 (0.18–1.92) |
| Type of consecutive night shifts |  |  |  |  |  |  |  |  |
| No night work | 1328/73 | Ref. |  | 1362/81 | Ref. |  | 1503/84 | Ref. |
| Only single night shifts | 38/3 | 0.96 (0.22–4.09) |  | 33/2 | 1.04 (0.24–4.49) |  | 19/3 | 3.42 (0.97–12.10) |
| Up to two consecutive night shifts | 132/10 | 1.34 (0.65–2.77) |  | 142/9 | 1.19 (0.57–2.44) |  | 91/9 | 1.44 (0.64–3.24) |
| ≥3 consecutive night shifts | 132/35 | **1.75 (1.12–2.73)** |  | 325/32 | **1.60 (1.01–2.54)** |  | 247/23 | **1.71** **(1.04–2.82)** |
| Frequency of ≥3 consecutive night shifts |  |  |  |  |  |  |  |  |
| 1–4 times | 283/22 | Ref. |  | 209/22 | Ref. |  | 167/19 | Ref. |
| 5–8 times | 57/7 | 1.78 (0.65–4.87) |  | 68/6 | 1.02 (0.35–2.97) |  | 43/2 | 0.41 (0.08–1.97) |
| >8 times | 29/6 | **3.43 (1.19–9.87)** |  | 48/4 | 1.02 (0.31–3.30) |  | 37/2 | 0.60 (0.124–2.93) |
| Frequency of ≥3 consecutive night shifts   (additionally, adjusted for no. of night shifts) |  |  |  |  |  |  |  |  |
| 1–4 times | 283/22 | Ref. |  | 209/22 | Ref. |  | 167/19 | Ref. |
| 5–8 times | 57/7 | 1.85 (0.55–6.21) |  | 68/6 | 0.62 (0.16–2.42) |  | 43/2 | 1.46 (0.19–10.99) |
| >8 times | 29/6 | 3.65 (0.80–16.65) |  | 48/4 | 0.45 (0.07–2.71) |  | 37/2 | 3.47 (0.32–37.43) |
| Average number of nights in a row |  |  |  |  |  |  |  |  |
| 1–1.5 nights | 76/5 | Ref. |  | 74/5 | Ref. |  | 40/5 | Ref. |
| 1.6–2.5 nights | 347/31 | 2.31 (0.67–7.90) |  | 317/26 | 1.14 (0.41–3.15) |  | 221/21 | 1.06 (0.34–3.35) |
| >2.5 nights | 114/12 | 2.96 (0.79–11.07) |  | 108/12 | 1.80 (0.59–5.45) |  | 94/9 | 1.05 (0.29–3.85) |
| Average number of nights in a row  (additionally, adjusted for no. of night shifts) |  |  |  |  |  |  |  |  |
| 1–1.5 nights | 76/5 | Ref. |  | 74/5 | Ref. |  | 40/5 | Ref. |
| 1.6–2.5 nights | 347/31 | 1.92 (0.54–6.85) |  | 317/26 | 1.04 (0.36–3.01) |  | 221/21 | 1.44 (0.44–4.65) |
| >2.5 nights | 114/12 | 2.24 (0.55–9.11) |  | 108/12 | 1.52 (0.45–5.06) |  | 94/9 | 1.57 (0.41–6.00) |
| Frequency of quick returns from night shift (<28 hours) |  |  |  |  |  |  |  |  |
| 1–8 times | 129/13 | Ref. |  | 299/22 | Ref. |  | 209/24 | Ref. |
| 9–18 times | 51/9 | 1.73 (0.80–3.72) |  | 84/13 | 2.17 (0.96–4.87) |  | 67/6 | 0.94 (0.35–2.53) |
| >18 times | 332/24 | **3.70 (1.50–9.11)** |  | 90/7 | 1.33 (0.52–3.41) |  | 68/2 | 0.341 0.074 1.567 |
| Frequency of quick returns from night shift (<28 hours)  (additionally, adjusted for no. of night shifts) |  |  |  |  |  |  |  |  |
| 1–8 times | 129/13 | Ref. |  | 299/22 | Ref. |  | 209/24 | Ref. |
| 9–18 times | 51/9 | **6.72 (1.79–25.16)** |  | 84/13 | 3.27 (0.86–12.41) |  | 67/6 | 3.27 (0.61–17.52) |
| >18 times | 332/24 | **50.49 (4.93–516.72)** |  | 90/7 | 3.24 (0.27–38.53) |  | 68/2 | 3.61 (0.17–76.25) |
| Long work shifts (≥10 hours) (any shift) |  |  |  |  |  |  |  |  |
| No long shifts (<10 hours) | 1199/64 | Ref. |  | 1211/74 | Ref. |  | 1402/74 | Ref. |
| 1–10 times | 397/30 | 1.26 (0.78–2.03) |  | 392/25 | 1.09 (0.67–1.77) |  | 277/37 | **2.50 (1.60–3.89)** |
| 11–20 times | 142/10 | 1.31 (0.63–2.72) |  | 116/11 | 1.37 (0.66–2.85) |  | 72/4 | 1.05 (0.37–2.99) |
| >20 times | 129/17 | **2.50 (1.38–4.51)** |  | 143/14 | 1.66 (0.88–3.13) |  | 109/4 | 0.73 (0.26–2.06) |
| Long working weeks (>40 hours) ever (all shift workers) |  |  |  |  |  |  |  |  |
| No long working weeks | 1566/109 | Ref. |  | 1618/107 | Ref. |  | 1709/103 | Ref. |
| Yes | 294/11 | 0.58 (0.30–1.11) |  | 235/17 | 1.09 (0.63–1.90) |  | 143/15 | **1.96 (1.10–3.51)** |

^a^Restricted to first births of women employed full time or part time during their whole pregnancy period, and preterm birth was dichotomized as <37 weeks and ≥37 weeks.
^b^Nurses including midwives, and Nursing assistants, (e.g. assistant nurses, careers, accommodation assistant, personal assistant).

**^c^**Adjusted for mother’s age (25–30 years (ref.), <25 years, 31–35 years, >35 years), mother’s height, BMI, smoking habits (yes, no (ref.)), education (higher education (university ≥3 years) (ref.), upper secondary/elementary or less), country of birth (Sweden (ref.), Nordic countries (except Sweden), Europe (except Nordic countries), other countries), and profession (midwives/nurses (ref.), nursing assistants).
However, smoking was not adjusted for 2 and ≥3 consecutive night shifts, average number of nights and quick returns because of missing data of smoking for cases in some categories.
**Bold** indicates statistical significance at *P* <0.05.
